# Aqueous Depolymerization of Polyethylene at Ambient Temperature: *In Situ* Generation of Permanganate Using Ozone

**DOI:** 10.1021/acssuschemeng.5c12302

**Published:** 2026-03-17

**Authors:** Michael S. Behrendt, Brandon D. Howard, Daniel Holmes, Scott Calabrese Barton, John R. Dorgan

**Affiliations:** † Chemical Engineering and Materials Science Department, 3078 Michigan State University, 3900 Collins Rd, Lansing, Michigan 48910, United States; ‡ Department of Chemistry, Michigan State University, 578 S. Shaw Lane, East Lansing, Michigan 48824, United States

**Keywords:** low-density polyethylene, aqueous oxidation, ozone, permanganate regeneration, chemical upcycling, green chemistry

## Abstract

The long sought-after goal of chemically recycling polyolefins at ambient temperature and pressure without the use of organic solvents is realized. Using ozone to regenerate a permanganate oxidant *in situ* in an aqueous environment, low-density polyethylene is converted to carboxylic acids and oligomeric wax. LDPE powder (with average particle sizes between 150 and 250 μm) serves as the substrate—reactions are conducted at atmospheric pressure and 30 °C. Water-soluble products are quantified using HPLC, with diacids having 4, 5, and 6 carbon units as the primary products. The remaining solid wax was analyzed for crystallinity by calorimetry (DSC), for acid number by titration, and for molecular functionality by ATR-IR and NMR spectroscopies. All three measured quantities increase with increasing reaction time. The acid number of the residual wax indicates a diacid carbon length of ∼30 (∼450 g/mol). Polymer oxidation occurs preferentially at side-chain branch points. Results suggest a two-reaction system in which branch-point tertiary carbons are selectively oxidized to yield linear carboxylic acids and ketones, followed by secondary depolymerization to yield water-soluble diacids. Experimental yields were 10% at 144 h.

## Graphical abstract

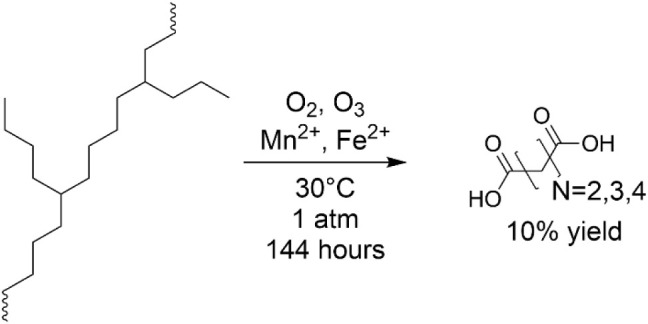

## Introduction

Novel recycling pathways are competing with the efficiency of well-established highly efficient processes that synthesize plastics from fossil fuels.^1^ Mechanical recycling can sometimes produce materials with degraded physical properties, leading to the need for various additives.^2,3^ Polymer additives, such as colorants and antioxidants, add additional separation or processing expenses.^4^ Most chemical recycling strategies adds a suite of additional expenses, including solvents, reactants, heat and electricity, chemically resistant equipment, separation and recovery steps, and loss to carbon dioxide.^1,3^ Chemically specific processes require collection, transport, and sorting to process a single polymer type; all other mass adds costs but no value. Multilayer films require additional delamination steps, and for this reason are typically limited to pyrolysis or incineration.^3,5,6^ While many chemical recycling processes are known, excessive energy requirements prohibit the praxis of most of these methods.

Chemical recycling of polyolefins typically requires either high temperatures or strong oxidants. Pyrolysis is an established method for converting polyolefins into reusable materials,^5^ and pyrolysis of polyolefins and mixed plastics as a chemical recycling method is well studied.^5,6^ Nitric acid can oxidatively break down polyolefins to dicarboxylic acids at a much lower temperature and energy use than pyrolysis,^7,8^ but is limited by environmental concerns and the production of undesirable side products. Although organic solvents have been tested as oxidant carriers,^9–11^ they introduce added cost and environmental risks. Similarly, high-temperature, high-pressure oxidation demands substantial capital and energy investment, limiting its practical viability even at temperatures considerably lower than those required for pyrolysis.^12,13^

This study introduces a novel pathway for the aqueous catalytic ozonation of low density polyethylene (LDPE) at ambient pressure and temperature, employing iron and manganese as cyclic radical initiators.^14^ This approach offers several advantages: water functions as the primary solvent, the metal catalysts are inexpensive and exhibit low toxicity, and the oxidation proceeds in a single step to yield value added products. Additionally, ozone as the primary oxidant can be generated on demand directly from air, eliminating the hazards associated with transporting or storing concentrated oxidants. These features have the potential to reduce both capital and operating costs compared to conventional chemical recycling strategies.

Dissolved ozone oxidizes Mn­(II) and Fe­(II) to higher oxidation states, generating transient permanganate species that selectively abstract hydrogen atoms from tertiary branch points in LDPE.^15–18^ The resulting tertiary radicals react with dissolved O_2_ to form peroxy intermediates, which drive the formation of ketone and carboxylate functionalities and ultimately induce chain scission.^19,20^ The free energy landscape of these reactions provides further insight into the proposed Mn/O_3_ redox cycle and associated oxidation pathways, which are summarized below.

The ozone/manganese/iron system has been extensively studied as a cyclic method for regenerating transient permanganate species. All water-soluble manganese oxidation states, except Mn^3+^, can react with ozone to regenerate permanganate at a pH of 0:^14^

1 $$Mn^{2+}+O_{3}\to MnO^{2+}+O_{2}$$

2 $$\Delta_{r}{G^{\prime }}_{298}=-106\;kJ\;mol^{-1}\left(pH=7\right)$$

3 $$MnO^{2+}+O_{3}+2H_{2}O\to MnO_{4}^{2-}+4H^{+}+O_{2}$$

4 $$\Delta_{r}{G^{\prime }}_{298}=-178\;kJ\;mol^{-1}\left(pH=0\right)$$

5 $$2MnO_{4}^{2-}\to MnO_{4}^{-}+MnO_{4}^{3-}$$

6 $$\Delta_{r}{G^{\prime }}_{298}=26\;kJ\;mol^{-1}\left(pH=0\right)$$

7 $$MnO_{4}^{3-}+O_{3}+2H^{+}\to MnO_{4}^{-}+O_{2}+H_{2}O$$

8 $$\Delta_{r}{G^{\prime }}_{298}=-317\;kJ\;mol^{-1}\left(pH=0\right)$$

While the disproportionation of $MnO_{4}^{2-}$ is not thermodynamically favored, it can progress with the rapid consumption of the products. Oxidation of Mn^3+^ by ozone is not thermodynamically favored in acidic environments.^14^

9 $$MnO^{2+}+Mn^{2+}+2H^{+}\to 2Mn^{3+}+H_{2}O$$

10 $$\Delta_{r}{G^{\prime }}_{298}=-2\;kJ\;mol^{-1}\left(pH=0\right)$$

11 $$Mn^{3+}+O_{3}+3H_{2}O\to MnO_{4}^{3-}+O_{2}+6H^{+}$$

12 $$\Delta_{r}{G^{\prime }}_{298}=102\;kJmol^{-1}\left(pH=0\right)$$

In an acidic environment, Mn^3+^ is expected to accumulate, and its disproportionation, influenced by complexation, becomes increasingly restricted as carboxylic acid products form. Fe^2+^ reacts rapidly with ozone to generate Fe^4+^, which can then participate in single electron transfer reactions with Mn^3+^, providing an alternative pathway for permanganate formation beyond direct oxidation or Mn^3+^ disproportionation. Additional manganese oxidation states are likely present only transiently; for example, MnO_2_ can dissolve under highly acidic, Mn^2+^ rich conditions through comproportionation with Mn^2+^.^14,21^ Ozone consumption in this system is inefficient: as reported by Reisz et al.,^14^ approximately 7.5 mol O_3_ are consumed per mole of MnO_4_^–^ produced due to competing reactions in which ozone acts as a reductant.^14,22^ Charge balance is maintained by the production or consumption of H^+^.

## Materials and Methods

### Safety

Ozone and powdered polymer both present an inhalation and explosion risk. All experiments should be conducted in a properly vented fume hood, and care should be taken to limit the exposure of polymer to oxidants to situations where the explosion risk is mitigated, such as an aqueous suspension.

### Chemicals

Glacial acetic acid was sourced from Supelco. Sulfuric acid was sourced from VWR Scientific at 95–98% purity. Oxygen gas cylinders were sourced from Airgas. Iron­(II) sulfate was sourced from Spectrum Chemical, assayed at 99.5–104.5% heptahydrate. Manganese acetate, malonic acid, and glutaric acid were sourced from Sigma-Aldrich at >99% purity. Succinic acid was sourced from TCI Co. at >99% purity. Adipic acid was sourced from Thermo Fisher Scientific at >99% purity.

### LDPE and Plastic Preprocessing Techniques

Virgin LDPE plastic (Agility 1021 LDPE) was donated by the Dow Chemical Company (Midland, MI). This is a grade with minimal additives. As-received pellets were milled and sieved to diameter, d, between 150 and 250 μm. The melt index (g/10 min @190 °C/2.16 kg) is 1.9 and was used to determine the molecular weight of ∼100 000 g/mol (calculations in ).^23^

### Control Experiments

Previous studies have examined relevant control conditions. Alter et al.^24^ investigated the aqueous depolymerization of polyethylene and found that ozone alone was ineffective over 24 h compared to oxidation with a manganese catalyst; our own extensive screening experiments corroborated this, showing no significant oxidation with ozone alone. They also observed the visible formation of Mn­(III–IV) solids, consistent with our preliminary experiments. Zawadiak et al.^13^ likewise found an increase in acid number in manganese-catalyzed aqueous systems using oxygen (without ozone). The difference between the manganese/iron system and manganese alone was not investigated in this work.

### Reactor System

A semibatch aqueous reactor with a 5 L disengagement vessel, a 3 L reactor vessel, and a Venturi ozone injection (A2Z Ozone A2Z S-6G) was used (piping diagram in Figure 1). Fluid was continuously circulated through the two vessels; as the fluid is pumped into the bottom of the reactor vessel, the fluid uses the Venturi effect to create suction, which is used to combine and disperse the liquid and gas streams. The gas and liquid streams were separated in the disengagement vessel, with the liquid recirculated and the offgas passed through an ozone stripping tank containing potassium iodide before venting to the fume hood.

**Figure 1:**
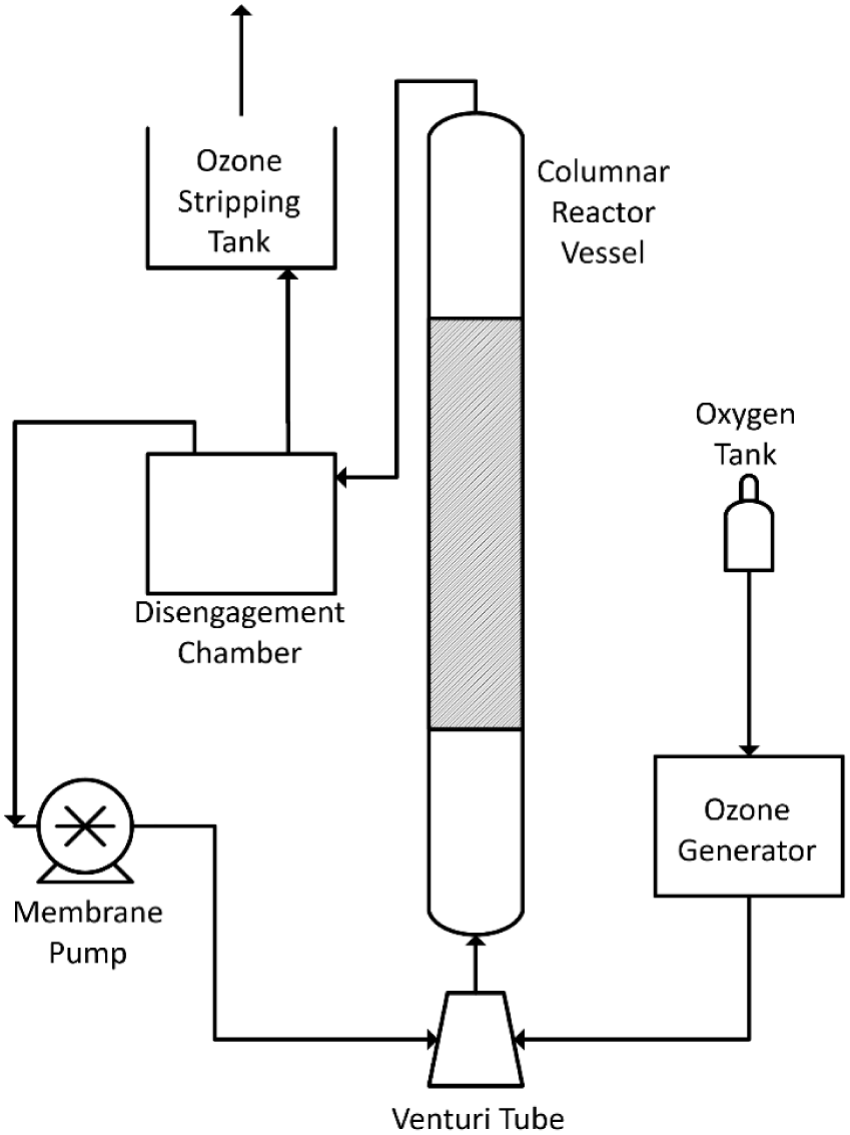
Diagram of the ozone reactor.

The reactor was charged as described in Table 1. Pump circulation and stirring of the disengagement chamber produced a stable suspension. Prior to ozone exposure, reactor fluid was equilibrated by bubbling O_2_ (1.0 L min^–1^) for 15 min. The ozonator was then activated at 100% power, producing a stream of approximately 3.5% ozone in oxygen. Samples (60 mL total mixed liquid/solid) were withdrawn approximately every 24 h. Each 60 mL sample was transferred to a scintillation vial; 2 mL of the aqueous portion was filtered through a 0.22 μm PTFE filter for HPLC analysis. The remaining slurry was vacuum filtered, washed with DI water to remove all catalyst species, and dried in a vacuum oven at 46 °C for ∼24 h, then ground and dried an additional 48 h under the same conditions to constant mass (<5% change). Solid were then analyzed by DSC, acid number titration, ATRIR, and ^13^C NMR.

**Table 1:** Standard Reaction Loading

Chemical	Quantity	Units	Purpose
Water	6000	ml	Reaction medium
Acetic Acid	600	ml	Radical Scavenger, Peroxyacid Carrier
Triton DF-12	1	ml	Surfactant for Initial LDPE Suspension
LDPE 150 < d < 250 μm	50	g	Reactant
Sulfuric Acid^a^	400	ml	Acidification, Solubilization of Manganese
Iron(II) Sulfate	0.565	μM	Cocatalyst
Manganese(II) Acetate	0.355	μM	Catalyst

a Under these conditions, the pH of the reactor fluid is −0.02.

### High-Performance Liquid Chromatography (HPLC)

High performance liquid chromatography (HPLC) was conducted on an Agilent 1200 Infinity Series using a Rezex ROA-Organic Acid H+ LC column 300 × 7.8 mm with 0.02N sulfuric acid in water as the mobile phase. One milliliter of liquid sample was run through the column for 45 min. Resulting chromatograms were compared to known samples calibrated for concentration.

13 $$\mathrm{Carbon}\mathrm{Yield}\;\left(\%\right)=\frac{IA_{HPLC,acid}\times Z_{acid}\times V\times MW_{CH_{2}}\times C{\#}_{acid}}{MW_{acid}\times g_{LDPE}}$$

where *IA*_*HPLC,acid*_ is the integrated area of the HPLC curve corresponding to the specific acid, *Z*_*acid*_ is the conversion factor from integrated HPLC area to acid concentration as calculated by standards, *V* is the reactor volume, $MW_{CH_{2}}$ is the molecular weight of a methylene group (14 g mol^–1^), *C*#_*acid*_ is the number of carbons in the acid of interest, *MW*_*acid*_ is the molecular weight of the acid of interest, and *g_LDPE_* is the mass of LDPE initially added to the reactor.

### Differential Scanning Calorimetry (DSC)

Differential scanning calorimetry (DSC) was conducted on a TA Instruments Q200. Samples were heated and cooled at a rate of 5 °C per minute. Crystallinity was determined by integrating the watts per gram versus time curve between the temperatures of 40 and 120 °C during the first heating.

14 $$\%\mathrm{Crystallinity}=\frac{IA_{\%c}}{\Delta H_{f,100\%}}\times 100\%$$

where *IA*_%*c*_ is the integrated area under the first melting endotherm in units of J/g, equivalent to the heat of fusion of the material (Δ*H*_*f*_). The known heat of fusion for 100% crystalline LDPE is Δ*H*_*f*,100%_ = 239 J g^–1^.^25^

### Acid Number Determination

Acid number was determined by titrating dissolved solid samples in mixed xylenes at near-reflux temperature with phenolphthalein as an indicator.^8^ The resulting liquid mixture was titrated with a standardized sodium hydroxide solution of 15% ethanol/85% mixed xylenes. Standardization was completed against a known quantity of succinic acid dissolved in DI water.

15 $$\frac{Carbon}{Acid}ratio=\frac{\left(g_{product}-V_{titrant}\times C_{titrant}\times MW_{COOH}\right)/MW_{CH_{2}}}{V_{titrant}\times C_{titrant}}$$

where *g_product_* is the grams of product used in the titration, *ml titrant* is the volume of titrant used, *V*_*titrant*_ is the volume of titrant used, *C*_*titrant*_ is the concentration of base in the titrant, *MW*_*COOH*_ is the molecular weight of a COOH group (45 g mol^–1^), and $MW_{CH_{2}}$ is the molecular weight of a CH_2_ group (14 g mol^–1^).

### C­{^1^H} NMR

^13^C­{^1^H} NMR was conducted on the recovered solid material dissolved in a solution of 0.05 M chromium acetylacetonate (Cr­(acac_3_))^26^ in 1,2,4-trichlorobenzene. The samples were previously washed with water using vacuum filtration to remove any traces of catalyst species, then dried to constant mass. Cr­(acac)_3_ is used to reduce ^13^C T_1_ relaxation time thereby increasing repetition rate and giving increased signal-to-noise, and shows no evidence of causing selective relative chemical shifts.^26^ Due to some small differences in shimming and concentration, spectra were aligned to the main short-chain methyl carbon signal at 14.12 ppm and normalized to the main backbone methylene peak at 30 ppm. NMR was conducted on a Bruker Avance III HD 500 MHz. NMR parameters included number of scans at 50000, recycle delay d1 = 1.0, and the zgpg30 pulse program.

### Attenuated Total Reflectance Infrared Spectroscopy (ATR-IR)

Attenuated total reflectance infrared spectroscopy (ATR-IR) was conducted using a Thermo Scientific Nicolet iS50 spectrometer. Spectra were modified using the ATR and baseline correction algorithms in the factory supplied software.

Savitzky–Golay smoothing of the data and its derivatives to identify differentiable peaks was conducted in MATLAB using a polynomial order of 5 and a frame size of 111 at approximately 4 data points per wavenumber. This frame size was selected arbitrarily from a series of frame sizes showing little change in target peak wavenumber.^27,28^

To calculate the carbonyl index, the spectral signal near the 1700 cm^–1^ area was baselined and deconvoluted in Origin graphing software using an additive Lorentzian and Gaussian distribution, specified in the software as PsdVoight1. Integrated areas were divided by known values for molar absorptivity (Table 2) to analyze changes in molar concentrations of various groups as a function of reaction time.

**Table 2:** Experimentally Determined Peak Positions and Literature Determined Extinction Coefficients

Assignment	Peak Position (cm^–1^)^b^	Extinction Coefficient (cm mmol^–1^)^a^
Carboxylic acids (isolated)	1738	16800
Ketones	1712	6880
Carboxylic acids (associated)	1696	16800

a Ref 29.

b Determined experimentally, substantially different than ref. 29

The Lambert–Beer law can be used to find the concentration of a sample from its IR spectra:^29^

16 $$C=\frac{IA}{\epsilon \times b}$$

Where *C* is the concentration, *IA* is the integrated area of the IR peak, ϵ is the molar absorptivity, and *b* is the path length. This quantitative relationship can be extended to functional groups using eq 5:

17 $$C_{N,x}=\frac{\frac{IA_{x}}{\epsilon_{x}}}{{\left(\frac{IA_{Acid,i}}{\epsilon_{Acid,i}}+\frac{IA_{Ketone}}{\epsilon_{Ketone}}+\frac{IA_{Acid,a}}{\epsilon_{Acid,a}}\right)}_{\max}}$$

Where *C*_*N*_,*_x_* is the normalized concentration of functional group x, *IA_x_* is the integrated area of the peak for functional group x, ϵ_*x*_ is the molar absorptivity of functional group x. The max subscript indicates that the maximum calculated value for the denominator is used for all concentration calculations, which causes all points to be normalized between 0 and 1. The subscripts *i* and *a* correspond to isolated and associated carboxylic acids, respectively.

## Results and Discussion

HPLC analysis (Figure 2) shows the carboxylic diacids containing four to six carbon atoms: succinic (C_4_), glutaric (C_5_), and adipic acid (C_6_) over 144 h of reaction time. Diacids with fewer than four carbons are known to react with manganese (II, III, and IV) to form carbon dioxide,^14,30,31^ while longer-chain diacids were not detected in appreciable amounts. C_4_ acid is the most stable dicarboxylic acid under radical oxidation conditions.^31,32^ Acetic acid is also expected to be present; however, it cannot be distinguished from the acetic acid introduced at the start of the reaction.

**Figure 2:**
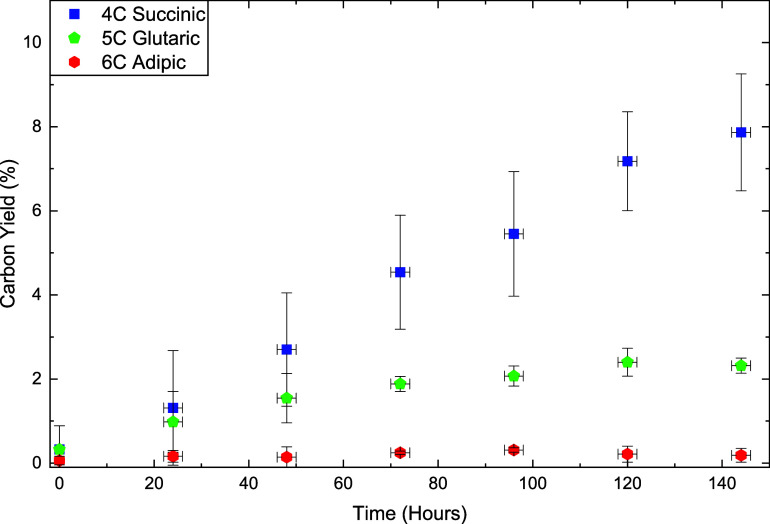
HPLC results for water-soluble products plotted against reaction time. No other chemicals were present in significant quantities. Error bars are ± SD from triplicate experiments.

Concentrations of soluble products follow trends indicating the constant breakdown of all dicarboxylic acids other than C_4_ acid. Yields of C_5_ and C_6_ acids increase initially but plateau around 48–72 h. C_3_ acid was tested in the reactor system and showed rapid degradation (see supplementary). C_2_ acid is known to react with some oxidation states of dissolved manganese,^14^ and Yang et al.^31^ demonstrated that both C_2_ and C_3_ acids exhibit high reaction rates with photooxidation-produced hydroxyl radicals.

A reaction is proposed to explain the formation of these products, shown in Scheme 1. Dicarboxylic acids longer than 4 carbons can react with hydroxy radicals to form peroxy radicals, which then perform an intramolecular ring formation to abstract a hydrogen from an α, β, or γ CH_2_ group.^19,31^ Yang et al.^31^ also proposed that short monocarboxylic acids undergo a similar reaction to form oxo- or hydroxy-substituted molecules favoring the ω position. In this system, ω-oxo- and ω-hydroxy monoacids will further react to dicarboxylic acids and can explain the absence of monocarboxylic acids.^33^ This reaction at the ω-site is expected to also occur at polymer chain ends that terminate in carboxylic acids; the steady rate of dicarboxylic acid production is consistent with a reaction that only occurs at chain ends, with the formation of each small diacid consuming and creating a single carboxylic acid group on the solid oligomer. Succinic acid represents an island of stability in oxidation systems^31^ and accumulates.

**Scheme 1:**
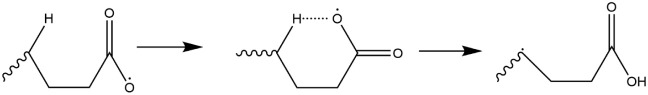
Zip Depolymerization to a Precursor for Autooxidation by a Carboxyl Radical

Figure 3 shows an increase in crystallinity as measured by DSC of the recovered solid phase starting at 45% and reaching about 70% after 72 h of reaction time. The final crystallinity value approaches the achievable maximum crystallinity of short, linear polyethylene chains, estimated at 80–85%.^34^ Amorphous regions are known to exhibit higher reactivity, which has been attributed to enhanced oxidant diffusion.^12^ Due to chain packing considerations, there may also be spatial correlation between amorphous domains and short-chain branch points, which would influence local reactivity. Notably, the crystallinity evolution deviates from that for succinic acid production. Crystallinity plateaus at approximately 72 h, whereas succinic acid continues to accumulate linearly. This suggests that crystallinity hinders but does not prevent the reaction. Crystallinity measurements can be augmented by acid titration which provides information on the changes to the end groups of the polymer molecules.

**Figure 3:**
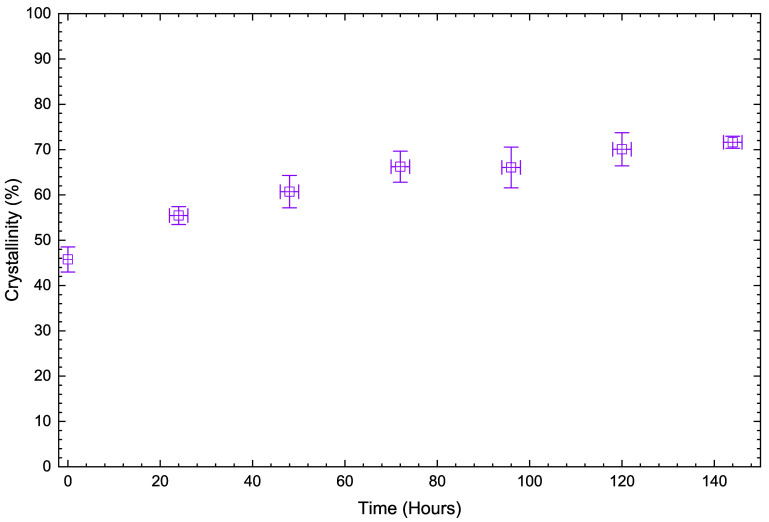
Crystallinity of the recovered solids as a function of reaction time. Error bars are ± SD from triplicate experiments.

Figure 4 shows the results from acid titration. The ratio of carbon atoms to carboxylic acid groups within the recovered solids is presented as a function of reaction time. Assuming the formation of linear monocarboxylic acids from branched LDPE, the corresponding chain length can be estimated as the value shown in Figure 4. The downward trend can be attributed to chain scission events that result in the formation of new carboxylic acid functionalities. The nonlinear trend observed during the early stages of the reaction suggests that oxidation occurs at points within the main chain, rather than exclusively at chain ends. The validity of the linear monoacid assumption is supported by NMR spectroscopy which confirms the sparse presence of methyl end groups and provides further insight by identifying branching patterns.

**Figure 4:**
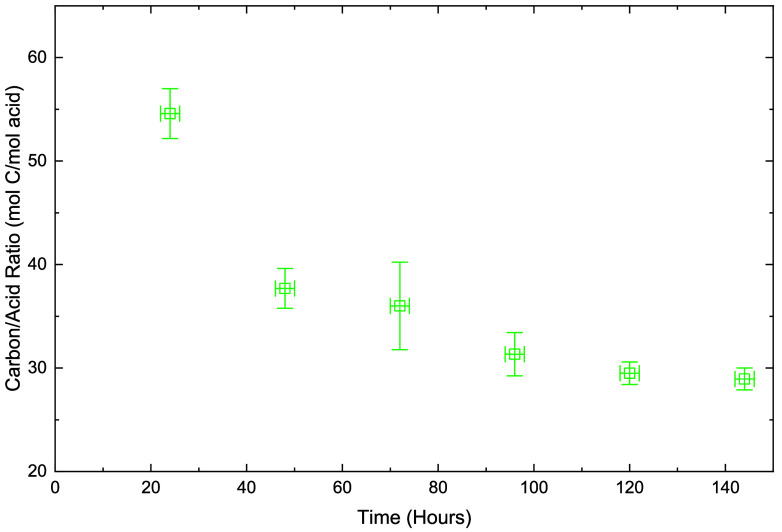
Carbon/carboxylic acid ratio as determined from acid titration versus reaction time. The point at 0 h was not included in this figure, as its value is effectively infinite. Error bars are ± SD from triplicate experiments.

^13^C­{^1^H} NMR and infrared (IR) spectroscopies were used to verify the presence of specific functional groups. NMR is superior to IR in distinguishing between esters and carboxylic acids because esters display a distinctive α-oxygen carbon signal at approximately 55–65 ppm.^35^ Such an ester peak is notably absent from the full range spectra shown in Figure 5. Peaks for alkene carbons, expected in the 100–140 ppm range, are likewise not visible as peaks or shoulders to the solvent peaks in that region. This absence confirms the oxidation process preferentially produces ketones and carboxylic acids, with minimal ester or alkene formation. Chromium acetylacetonate appears as two minor peaks marked in Figure 5 and solvent signals for 1,2,4-trichlorobenzene are clustered near 130 ppm. Two broad peaks consistent with the formation of ketones (208 ppm) and carboxylic acids (179 ppm) are observed in the oxidized material. In contrast, the virgin material shows no evidence of oxidation. The presence of ketone and carboxylic acid functionalities enables interpretation of methylene group signals, which offer the most profound structural characterization of chain structure.

**Figure 5:**
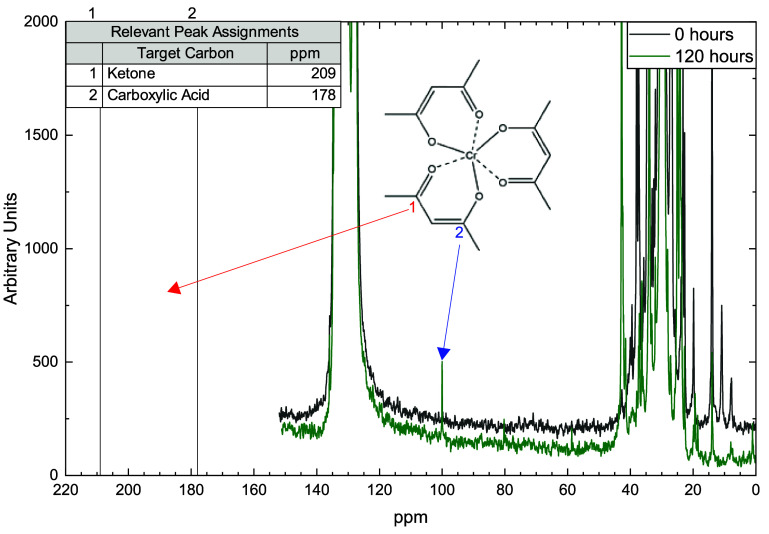
^13^C­{^1^H} NMR spectra of virgin (0 h) and recovered solids (120 h) after oxidation.

The NMR spectral region spanning 10–44 ppm for alkanoic carbons in virgin material is shown in Figure 6. Branch points (39.64 and 38.14 ppm) and their associated α- and β-carbons (34.53 and 27.25 ppm respectively) are clearly resolved in the spectra. While peak overlap is common among various short-chain branches, ethyl branches exhibit distinct C1 signals (11.07 ppm), and *n*-butyl groups show differentiated C2 peaks (23.36 ppm), allowing for the observation of separate trends from those of longer branches.^36^ LDPE molecules are estimated to contain approximately 5–15 *n*-butyl branches, along with 1–2 each of ethyl, *n*-amyl, *n*-hexyl, and other long-chain branches for each 1000 polymer carbons, with trace levels of methyl and *n*-propyl substitutions.^37^ Accordingly, oxidation is expected to produce four new peaks, one each for α-ketone, β-ketone, α-carboxylic acid, and β-carboxylic acids. The aforementioned intramolecular ring-forming reaction that produces water-soluble diacids would produce an acid group for each acid group consumed and would not resolve in the NMR spectra. As most polyolefin depolymerization and water treatment chemistries are contingent on the production of carbon radicals,^20^ radical chemistry is expected to be responsible for the development of functional groups on the carbon backbone of the solid material. Carbon radicals have maximum stability on tertiary carbons,^33^ so radical initiation is expected to occur primarily at short-chain branch points, which is confirmed by NMR.

**Figure 6:**
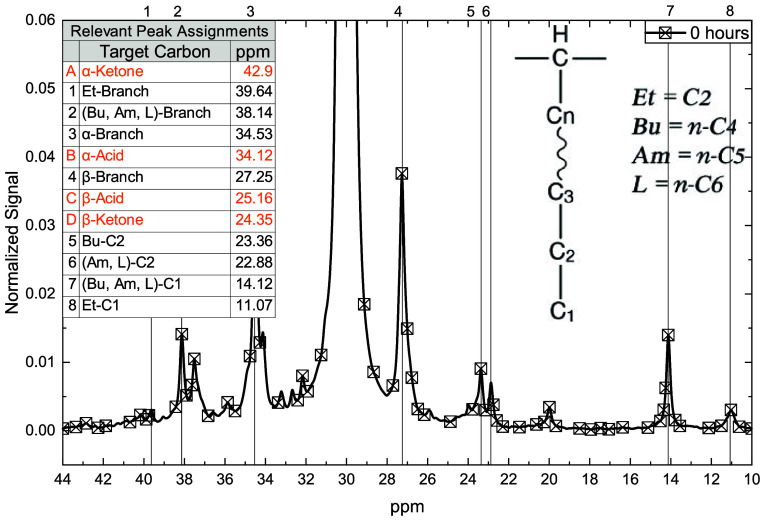
^13^C­{^1^H} NMR spectra of virgin material focused on the peaks relevant to methylene, as well as those associated with branching. Peaks were identified from literature sources.^36^

Figure 7 shows the ^13^C­{^1^H} NMR spectra of oxidized material and the development of changes in the alkanoic environment for (7.A) branch points and α-functionalization, (7.B) C2 and β-functionalization, and (7.C) C1 carbons in descending order of ppm. These combined spectra affirm the development of α- and β-carbonyl carbon peaks and the near complete elimination of branch points. Figure 7A shows all branch points’ peaks (39.64 and 38.14 ppm) significantly decrease for all chains by the 48-h mark. This change is accompanied by parallel reductions in the signals of α-carbons (34.53 ppm) adjacent to these branches. In contrast, signals for α-ketone carbons (42.90 ppm) show little change after 24 h, indicating a steady-state concentration of the ketone group. α-Carboxylic acid signals (34.12 ppm) reach steady-state at approximately 72 h. A small α-branch signal persists throughout (34.53), regardless of reaction extent, possibly from unreacted branch points in the interior of crystalline areas. Figure 7B highlights the methylene region associated with β-branch (27.25 ppm), β-acid (25.16 ppm), and β-ketone peaks (24.35 ppm), all of which follow accordant trends consistent with oxidation at branch points. Figure 7B can also be used to distinguish Bu branches (23.36 ppm) from Am and L branches (both 22.88 ppm) by the C2 peak. Bu branches are eliminated by 48 h while a portion of Am and L branches persist throughout the entire period of oxidation. With little difference in reactivity expected between Bu and Am branches, this residual signal is primarily attributed to long-chain branching. Figure 7A confirms some residual branching, showing distinct signals for (Bu, Am, L)-C1 peaks (14.12 ppm) partially persisting after oxidation while ethyl-C1 peaks (11.07 ppm) are eliminated. Together, these NMR spectra support the hypothesis that initial oxidation preferentially occurs at short-chain branch points, decomposing them into acid and ketone functionalities.

**Figure 7:**
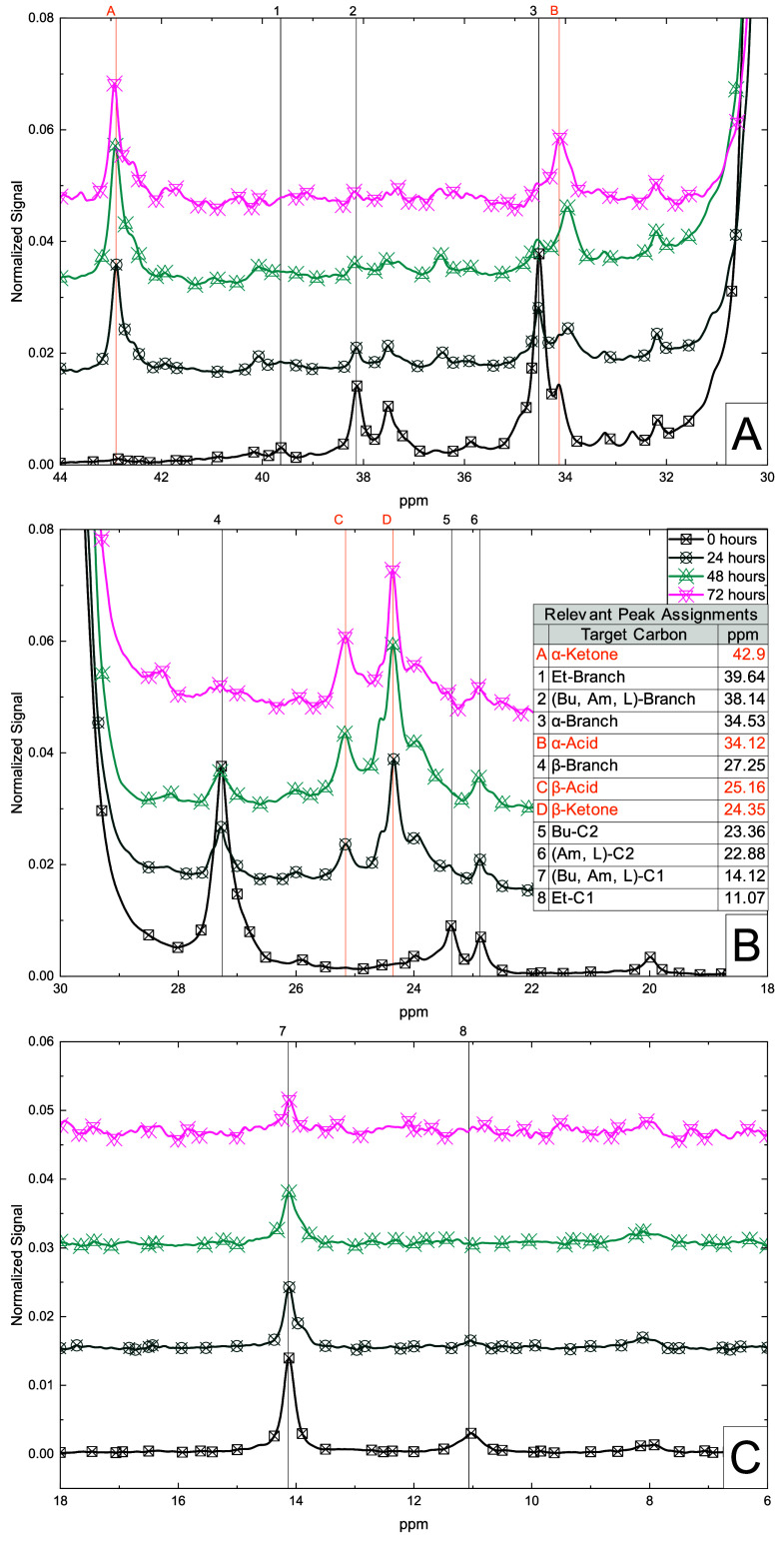
^13^C­{^1^H} NMR spectra of virgin, 24, 48, and 72 h oxidized material focused on the (A) 44–30 ppm, (B) 30–18 ppm section, and (C) 18–6 ppm section of the peaks relevant to methylene, as well as those carbons associated with branching.

Based on the NMR results, a partial mechanism is presented in Scheme 2. The elimination of short branch points at the 72-h mark indicates the polymer must be divided into oligomers having a length corresponding to the original average distance between branch points. When a β-scission event occurs at a secondary carbon, the result is an aldehyde and a primary carbon radical, both of which are expected to rapidly react with oxidants in the system to form carboxylic acids. At a tertiary carbon, β-scission necessitates the formation of a ketone, either on the polymer backbone (when the scission occurs on the short chain), or at a distance from the new chain end equal to the length of the short chain (when the scission occurs at the polymer backbone). Ketones are relatively stable against oxidation and represent deactivation toward further product formation. Tertiary carbon scission proceeds through the formation of the most stable radical,^38^ indicating backbone scission is the favored route. Based on the known branching structure of LDPE, this backbone mechanism should produce an acid:ketone ratio between 0.66:1 and 1:1. IR was used to determine the molar ratios of these groups for our process and a ratio of approximately 0.60:1 was found. As this value falls outside the expected range for a purely tertiary scission-based process, parallel reactions must be present; for example, the termination of primary radicals through cross-linking rather than oxidation, or scission events at secondary carbons. Secondary carbon scission events must contribute, as permanganate is known to react with HDPE.^18,39^

**Scheme 2:**
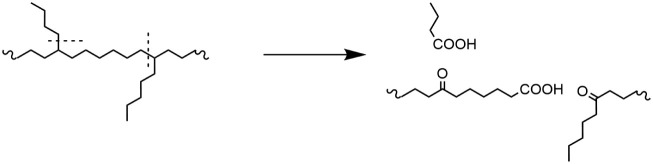
Results of the 2 Possible β-Scission Directions Resulting from Tertiary Carbon Autooxidation

ATR-IR spectroscopy was used to determine relative amounts of functionalization in the region centered around 1710 cm^–1^, corresponding to a convolution of signals from several carbonyl-containing groups. These functional groups include ketones, aldehydes, esters, carboxylic acids, and various carbonyl groups with adjacent unsaturation.^29^ Given NMR evidence controverting unsaturation and ester formation, the measured IR signals are attributed exclusively to ketones and carboxylic acids. To enhance the resolution of the convoluted carbonyl IR signals, the fourth derivative and the inverted second derivative of the spectrum are plotted in Figure 8A.^27,28,40^ Coincident maxima in these derivative plots suggest the presence of distinct carbonyl environments.^28^ The IR absorption frequencies of carboxylic acids are known to vary based on factors including hydrogen bonding, crystallinity, and dimerization.^41^ Consequently, the IR spectrum exhibits one ketone peak and two distinct carboxylic acid peaks, with variations in the local chemical environment producing minimal shifts in their absorption positions. This interpretation is corroborated by washing the solid product with a basic solution which deprotonates acid-specific peaks and changes their frequencies, then repeating the ATR analysis (see supplemental). This carbonyl distribution conclusion contradicts the work of several authors investigating similar methods of polyethylene oxidation^12,13,40,42^ but affirms other carefully conducted studies.^43^ Figure 8B shows the deconvolution of the carbonyl region to produce separate peaks for quantification through integration, a good fit to the experimental signal and consistent alignment with the peaks from derivative spectroscopy is obtained. The areas of the deconvoluted peaks can be integrated to reveal the relative molar concentrations of the functional groups.

**Figure 8:**
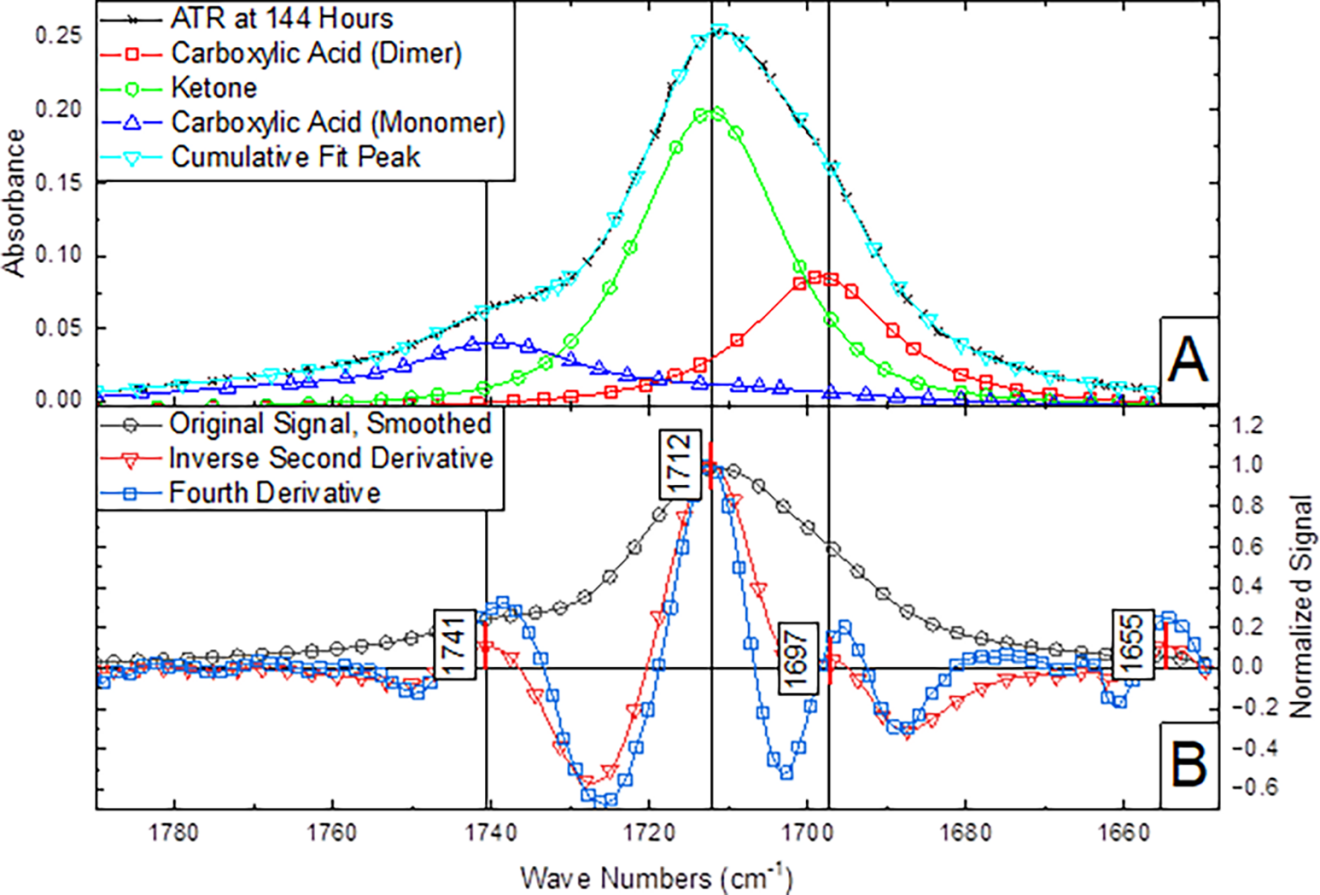
(A) Normalized Savitzky–Golay smoothed ATR spectra of LDPE oxidized for 144 h plotted alongside the fourth and inverse second derivative. (B) IR-ATR spectra of the carbonyl region for oxidized LDPE spectra after 144 h of oxidation deconvoluted into peaks corresponding to differentiable functional groups.

Figure 9 presents the change in carbonyl concentration over time. These values are determined by using the deconvolution method demonstrated in Figure 8B by dividing the integrated area by the molar absorptivity (values shown in Table 2) and normalized to the maximum total concentration at the final time point. Signals saturate at the 48-h mark, a result that deviates slightly from NMR results. NMR and acid number detection were conducted under melt conditions, while ATR-IR is reliant on an evanescent wave through the solid material and is subsequently only able to observe oxidation over a depth of about 1 μm. Radicals in the carbon backbone can diffuse via intra- or intermolecular reactions within amorphous regions where oxygen is present,^12,13^ allowing for penetration into regions of the solid polymer particle. The backbiting zip depolymerization reaction conversely requires mobility around a chain end and is unlikely to occur in internal areas where chain movement is restricted. IR reveals that, despite NMR evidence that radical debranching reactions continue to occur until 72 h, the quantity of surface-accessible acid groups changes little after 24 h, which reconciles the difference in trends between the formation of soluble products and the elimination of branch points. With the addition of details on the cyclic regeneration of the catalysts, a complete reaction mechanism is now proposed.

**Figure 9:**
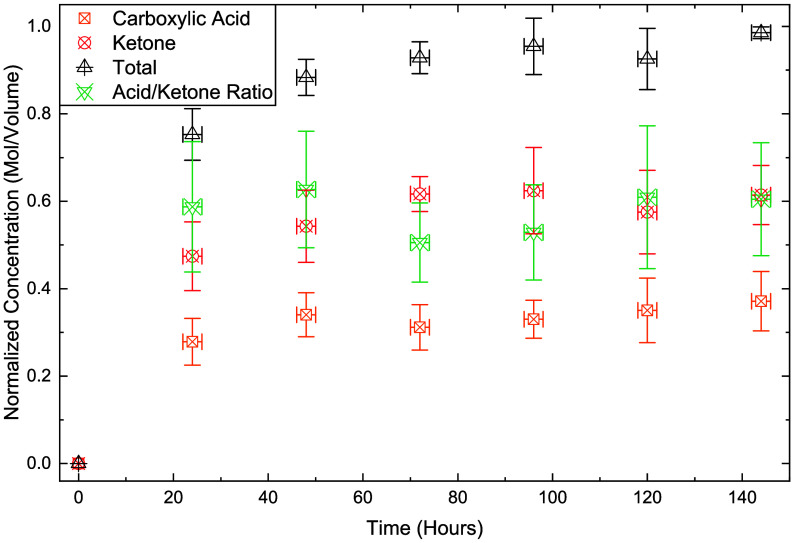
Normalized concentration of carboxylic functional groups on recovered solid material compared to reaction time using ATR data. Integrated areas are divided by molar absorptivity prior to normalization. Error bars are ± SD from triplicate experiments.

We assume that ozone dissolves in the reactor fluid and oxidizes the manganese and iron catalysts to push the equilibrium toward permanganate.^14^ Permanganate exhibits the uncommon capability to engage in hydrogen atom abstraction chemistry despite not being a radical species, enabling it to remove a hydrogen atom from a tertiary branch point within the polymer backbone.^15–18^ This tertiary carbon radical reacts with dissolved oxygen to initiate an autoxidation pathway by creating a main chain scission event, resulting in a ketone and a carboxylic acid. Terminal carboxylic acids can form radicals through decomposition of a hydroperoxide or reaction of a primary carbon radical with oxygen^19^ which then abstract an intramolecular hydrogen in a zip depolymerization step. Following the well-understood autoxidation pathway, the resulting secondary carbon radical creates another chain scission event.^20^ In this oxidative reaction, two carboxylic acids form at the scission point. Once a radical is formed at a secondary or tertiary carbon, it can either react with oxygen to begin the autoxidation process or abstract a neighboring hydrogen, effectively migrating down the polymer chain and initiating autoxidation at a site internal to the polymer particle.^20^ Once all tertiary branch points have reacted, radical initiation is limited to initiation from carboxylic acid end groups. The solid material consists of insoluble oligomers of a length comparable to the distance between original branch points, terminating primarily in carboxylic acids or butyl ketones. Once surface-accessible acid groups are developed, soluble products can form via the zip depolymerization reaction.^19^

## Conclusions

This work realizes the goal of chemically oxidizing polyolefins at ambient temperature and pressure without the use of organic solvents. Depolymerization of low-density polyethylene was conducted at 30 °C and atmospheric pressure to produce linear long-chain dicarboxylic acids of an average carbon length of ∼30. Soluble dicarboxylic acids were produced as a secondary product in yields of up to 10% for the four-carbon diacid (succinic acid).

A two-reaction system is proposed in which branch point tertiary carbons are oxidized by permanganate to produce linear diacids and some ketones. Subsequent short diacids are formed by repeated backbiting hydrogen abstraction from a carboxylic acid radical. This mechanism is supported by NMR evidence showing the decrease in detected branch points commensurate with the formation of ketone and carboxylic acid functionalities in the solid material. Short diacid production rate is not affected by the depletion of polymer branch points, and is consistent with a reaction rate reliant on surface-available functional groups detected using IR.

These reactions reveal a decisive step toward an economically competitive chemical recycling process by eliminating the need for high-temperature or high-pressure equipment and organic solvents. From an environmental, health, and safety perspective, using only air and electricity positions this approach favorably compared to other strong oxidants or organic solvent systems.

## Supplementary Material





## Notes

(PDF) PDF file containing supplemental experiments and expanded reaction scheme
MATLAB code for Savitzky–Golay smoothing, raw data in .xlsx form for ATR, DSC, and NMR for the three repeat experiments, and a README file explaining what data is included (ZIP)
