# Supplementary material for: Aqueous Depolymerization of Polyethylene at Ambient Temperature: In Situ Generation of Permanganate Using Ozone
Source: ACS Sustain Chem Eng. 2026 Mar 17;14(12):5995–6005. doi: 10.1021/acssuschemeng.5c12302 (PMC13040521; doi:10.1021/acssuschemeng.5c12302)
Supplement: Supplementary file 1 [file sc5c12302_si_001.pdf]

Supporting Information

AQUEOUS DEPOLYMERIZATION OF POLYETHYLENE AT AMBIENT TEMPERATURE: *IN SITU* GENERATION OF PERMANGANATE USING OZONE.

Authors:
Michael S. Behrendt,[1]* Brandon D. Howard[1], Daniel Holmes[2], Scott Calabrese Barton[1], John R. Dorgan,[1]*

Affiliations:

[1]Chemical Engineering and Materials Science Department, Michigan State University, 3900 Collins Rd., Lansing, MI 48910, USA

[2]Department of Chemistry, Michigan State University, 578 S. Shaw Lane, East Lansing, MI 48824, USA

*Corresponding authors:

Michael S. Behrendt, behren23@msu.edu, Phone (517) 928-4092

John R. Dorgan, JD@msu.edu

Supporting information contains:

Number of pages: 11

Number of figures: 3

Number of tables: 3

Number of schemes: 3

Table of Contents

Supplementary Experiments: S4
Base-washed solid material S4
Diacid Stability Tests S5
Effect of Catalyst Loading S6
Expanded Reaction Description S7

List of Figures and Tables:

1. Figure S1: Normalized absorbance versus wave number for 144 hours oxidized solid material compared to the same material altered by washing with dilute sodium hydroxide solution or methyl esterification.
2. Table S1: Reaction loading to test breakdown of diacids
3. Figure S2: Analysis of the breakdown of diacids by ozonolysis.
4. Table S2: Reaction conditions to test the effects of increasing catalyst concentration.
5. Figure S3: Diacid yield versus time for an experiment using higher (10x) catalyst loading.
6. Scheme S1: Hydrogen abstraction by permanganate
7. Scheme S2: Autooxidation reactions occurring at a tertiary carbon. n-butyl is chosen as the most common short chain branch in LDPE.
8. Scheme S3: Zip depolymerization to a precursor for autooxidation by a carboxyl radical

LDPE preprocessing

Virgin LDPE (Agility 1021, Dow Chemical) was donated by Dow Chemical (Midland, MI). The grade has minimal additives. As-received pellets were granulated (Hellweg Maschinebau MRS-H) and sieved using ASTM E-11 standard testing sieves (Fisher Scientific): 1.18 mm, 250 μm, and 150 μm. Powder with particle diameter d between 150 μm and 250 μm was used for all experiments.

Molecular weight was calculated from the melt flow index (MFI) using the following equations:[1]

$$log\eta_0 = -1.2855 * logI_2 + 4.3546 = \ 3.998$$

$$M_w = 10^{\frac{log\eta_0 + 16.343}{4.0573}} = \ 103{,}141 \ g\ mol^{-1}$$

Where η_0 is zero-shear viscosity, I_2 is MFI (1.9 g/10 min,(ASTM D1238)), and M_w is molecular weight.

Supplementary Experiments:

Base-washed solid material

Recovered solids were treated either (i) with dilute NaOH (aq) for 24 h to deprotonate surface-accessible carboxylic acids or (ii) refluxed in MeOH with catalytic H2SO4 at 100 °C for 24 h to promote esterification. Normalized ATR-IR spectra (Figure S2) show a persistent ketone peak at 1714 cm^{-1} after both treatments; carboxyl peaks (~1698 and 1739 cm^{-1}) were eliminated or esterified as expected. These data support the assignment of the 1698 and 1739 cm^{-1} features to associated and isolated carboxylic acids, respectively. The IR results are shown in figure S1, focused on the region relevant to carboxyl functionalities.

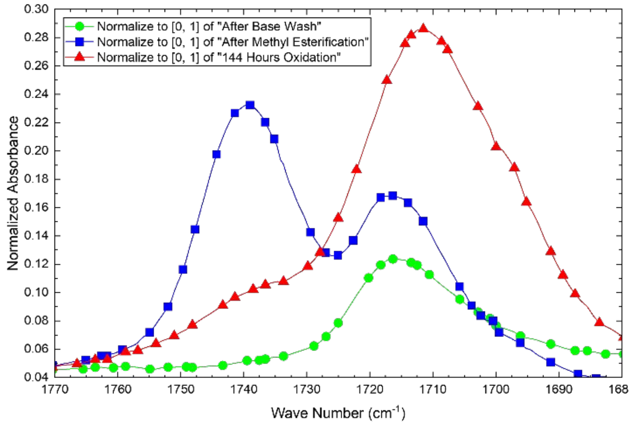

Figure S1: Normalized absorbance versus wave number for 144 hours oxidized solid material compared to the same material altered by washing with dilute sodium hydroxide solution or methyl esterification.

Diacid Stability Tests

To investigate diacid stability, known amounts of selected diacids were added to the reactor at t = 0 (loading conditions in Table S1). Resulting quantities as determined by HPLC are plotted against time are shown in Figure S2 and quantify breakdown rates under the reactor conditions.

Table S1: Reaction loading to test breakdown of diacids

Chemical	Quantity	Units	Purpose
Water	6000	ml	Reaction medium
Acetic Acid	600	ml	Radical Scavenger, Peroxyacid Carrier
Triton DF-12	1	ml	Surfactant for Initial LDPE Suspension
LDPE 150<d<250 μm	50	g	Reactant
Sulfuric Acid	400	ml	Acidification, Solubilization of Manganese
Iron (II) Sulfate	5.65	μM	Cocatalyst
Manganese (II) Acetate	3.55	μM	Catalyst
Adipic Acid	12.5	g	Test Material
Glutaric Acid	12.5	g	Test Material
Succinic Acid	12.5	g	Test Material
Malonic Acid	12.5	g	Test Material

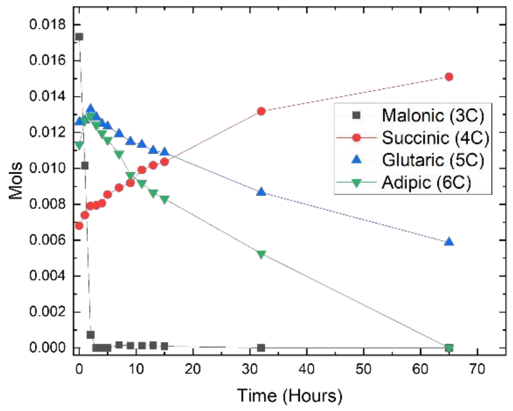

Figure S2: Analysis of the breakdown of diacids by ozonolysis.

Effect of Catalyst Loading

Tests with 10x catalyst loading (Table S2) produced increased steady-state concentrations of longer diacids (Figure S3). These observations are consistent with stronger complexation of carboxylates to higher Mn oxidation states (e.g., MnO^{2+} complexes) that slow further oxidation to smaller diacids.

Table S2: Reaction conditions to test the effects of increasing catalyst concentration.

Chemical	Quantity	Units	Purpose
Water	6000	ml	Reaction medium
Acetic Acid	600	ml	Radical Scavenger, Peroxyacid Carrier
Triton DF-12	1	ml	Surfactant for Initial LDPE Suspension
LDPE 150<d<250 µm	50	g	Reactant
Sulfuric Acid	400	ml	Acidification, Solubilization of Manganese
Iron (II) Sulfate	5.65	µM	Cocatalyst
Manganese (II) Acetate	3.55	µM	Catalyst

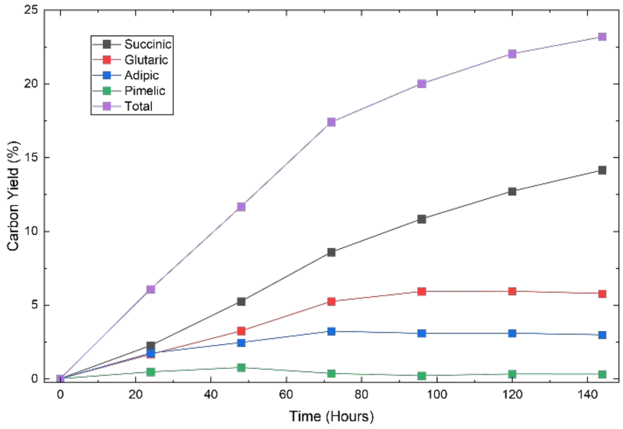

Figure S3: Diacid yield versus time for an experiment using higher (10x) catalyst loading.

Expanded Reaction Description

We propose that dissolved ozone oxidizes Mn(II) and Fe(II) to higher oxidation states, shifting equilibrium toward transient permanganate species that can abstract hydrogen atoms at tertiary branch points on LDPE.[2-5] This tertiary radical reacts with O_2 to form peroxy intermediates that lead to ketone and carboxylate functionalities and chain scission.[6-7] Hypothesized reaction steps can be examined in greater detail through a consideration of the free energy of reactions. Subsequent intramolecular processes and β-scission events yield short dicarboxylic acids through zip depolymerization (Scheme S3). The hypothesized Mn/O_3 redox cycle and representative oxidation steps are summarized below:

The ozone/manganese/iron system has been extensively studied as a cyclic method for regenerating transient permanganate species. All water-soluble manganese oxidation states, except Mn^{3+}, can react with ozone to regenerate permanganate:[8]

$$Mn^{2+} + O_3 \rightarrow MnO^{2+} + O_2 \quad \text{(S1)}$$
$$\Delta_r G'_{298} = \ -106\ kJ\ mol^{-1} \quad \text{(S2)}$$

$$MnO^{2+} + O_3 + 2H_2O \rightarrow MnO_4^{2-} + 4H^+ + O_2 \quad \text{(S3)}$$
$$\Delta_r G'_{298} = \ -178\ kJ\ mol^{-1} \quad \text{(S4)}$$

$$2MnO_4^{2-} \rightarrow MnO_4^- + MnO_4^{3-} \quad \text{(S5)}$$
$$\Delta_r G'_{298} = \ 26\ kJ\ mol^{-1} \quad \text{(S6)}$$

$$MnO_4^{3-} + O_3 + 2H^+ \rightarrow MnO_4^- + O_2 + H_2O \quad (S7)$$
$$\Delta_r G'_{298} = -317\ kJ\ mol^{-1} \quad (S8)$$

While the disproportionation of MnO_4^{2-} is not thermodynamically favored, it can progress with the rapid consumption of the products. The Mn^{3+} species can be produced via reactions that compete with the desired cyclic permanganate regeneration. Oxidation of Mn^{3+} by ozone is not thermodynamically favored and limited by the saturated oxygen content in the liquid phase:

$$MnO^{2+} + Mn^{2+} + 2H^+ \rightarrow 2Mn^{3+} + H_2O \quad (S9)$$
$$\Delta_r G'_{298} = -2\ kJ\ mol^{-1} \quad (S10)$$

$$Mn^{3+} + O_3 + 3H_2O \rightarrow MnO_4^{3-} + O_2 + 6H^+ \quad (S11)$$
$$\Delta_r G'_{298} = 102\ kJ\ mol^{-1} \quad (S12)$$

In a scenario using these reactions, Mn^{3+} would accumulate and eventually quench the reaction. However, Reisz et al.[8] investigated iron as a cocatalyst, and hypothesized that mixed metal oxides of Mn^{3+} could potentially have a route to oxidation by ozone. The ozone/manganese/iron system enables continuous regeneration of active manganese species. MnO_2 forms a rutile colloid and is highly resistant to further oxidation; The interaction of anions with Mn(IV) plays a crucial role in regulating the equilibrium among manganese oxidation states.[8]

Once permanganate is introduced into the system, it can initiate oxidation at the LDPE surface via a well-documented mechanism. Permanganate exhibits a unique ability to abstract hydrogen atoms from organic substrates without itself exhibiting radical character and is demonstrated in Scheme S1.[2-5] This hydrogen abstraction is thermodynamically favorable due to the formation of a strong Mn–OH bond.[4]

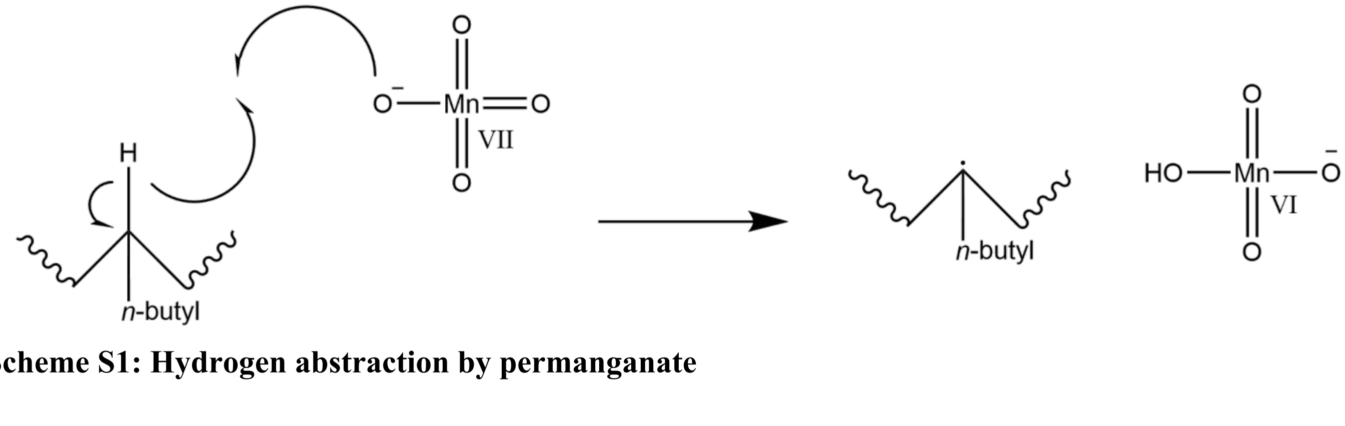

Scheme S1: Hydrogen abstraction by permanganate

Once a radical is formed at a short chain branch point, it can either react with oxygen to begin the autooxidation process or abstract a neighboring hydrogen, effectively migrating down the

polymer chain and initiating autooxidation at a site internal to the polymer particle.[7] Autooxidation produces a ketone and a carboxylic acid group for each scission event; the scission point is expected to favor main chain scission (scission B in Scheme S2).[9] Once all tertiary branch points have reacted, scission at these points can no longer occur. The solid material consists of insoluble oligomers of a length comparable to the distance between original branch points, terminating in carboxylic acids or butyl ketones. Once surface-accessible acid groups are developed, soluble products can form via a zip depolymerization reaction.

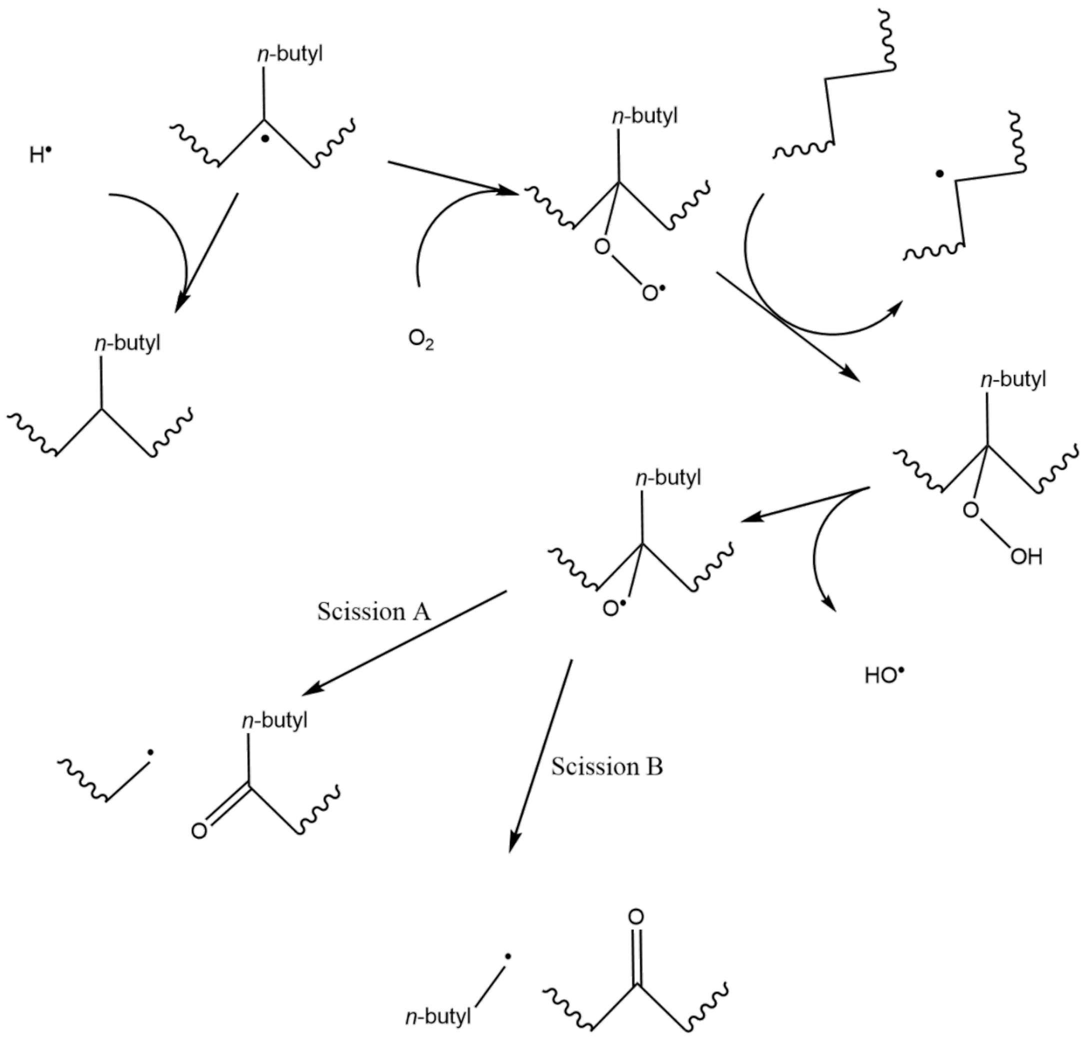

Scheme S2: Autooxidation reactions occurring at a tertiary carbon. n-butyl is chosen as the most common short chain branch in LDPE.

The zip depolymerization pathway occurring at functionalized oligomer chain ends, shown in Scheme S3, begins with the initialization of a carboxylate radical, which is then able to abstract an intramolecular hydrogen at a distance commensurate with stable ring formation. Initial radicals may originate through the decomposition of peroxyacids or through the reaction of

primary carbon radicals with molecular oxygen.[6] The direction of the proceeding β-scission will favor the creation of the more stable primary carbon radical attached to the longest available chain.[9] This β-scission dynamic as well as the stability of six-membered rings indicate succinic acid will be the primary product, with other short diacids as secondary products.

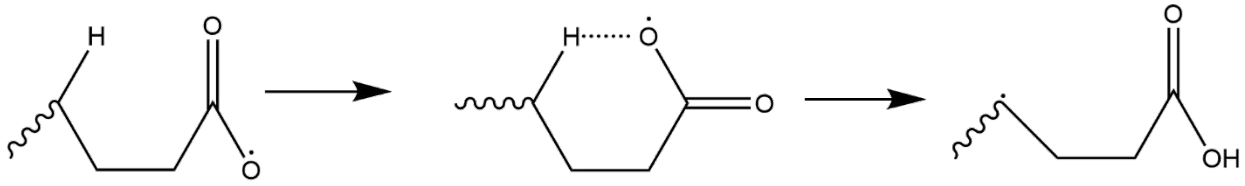

Scheme S3: Zip depolymerization to a precursor for autooxidation by a carboxyl radical

1. Zhu, K.; Wu, D.; Yang, S.; Cao, C.; Zhou, W.; Qian, Q.; Chen, Q. Identification of Aged Polypropylene with Machine Learning and Near–Infrared Spectroscopy for Improved Recycling *Polymers* [Online], 2025, p. 700.
2. Gardner, K. A.; Kuehnert, L. L.; Mayer, J. M., Hydrogen Atom Abstraction by Permanganate: Oxidations of Arylalkanes in Organic Solvents. *Inorg Chem* **1997,** *36* (10), 2069-2078.
3. Strassner, T.; Houk, K. N., Mechanism of Permanganate Oxidation of Alkanes: Hydrogen Abstraction and Oxygen "Rebound". *Journal of the American Chemical Society* **2000,** *122* (32), 7821-7822.
4. Mayer, J. M., Hydrogen Atom Abstraction by Metal−Oxo Complexes: Understanding the Analogy with Organic Radical Reactions. *Accounts of Chemical Research* **1998,** *31* (8), 441-450.
5. Fávaro, S. L.; Rubira, A. F.; Muniz, E. C.; Radovanovic, E., Surface modification of HDPE, PP, and PET films with KMnO4/HCl solutions. *Polymer Degradation and Stability* **2007,** *92* (7), 1219-1226.
6. Karlsson, S.; Hakkarainen, M.; Albertsson, A. C., Dicarboxylic acids and ketoacids formed in degradable polyethylenes by zip depolymerization through a cyclic transition state. *Macromolecules* **1997,** *30* (25), 7721-7728.
7. Schnabel, W., *Polymer Degradation: Principles and Practical Applications*. De Gruyter: 1981.
8. Reisz, E.; Leitzke, A.; Jarocki, A.; Irmscher, R.; von Sonntag, C., Permanganate formation in the reactions of ozone with Mn(II): a mechanistic study. *Journal of Water Supply Research and Technology-Aqua* **2008,** *57* (6), 451-464.
9. Bietti, M.; Gente, G.; Salamone, M., Structural effects on the beta-scission reaction of tertiary arylcarbinyloxyl radicals. The role of alpha-cyclopropyl and alpha-cyclobutyl groups. *J Org Chem* **2005,** *70* (17), 6820-6.
